# Winged Pea Aphids Can Modify Phototaxis in Different Development Stages to Assist Their Host Distribution

**DOI:** 10.3389/fphys.2016.00307

**Published:** 2016-08-02

**Authors:** Yi Zhang, Xing-Xing Wang, Xiangfeng Jing, Hong-Gang Tian, Tong-Xian Liu

**Affiliations:** ^1^State Key Laboratory of Crop Stress Biology for Arid Areas, College of Plant Protection, Northwest A&F UniversityYangling, China; ^2^Key Laboratory of Integrated Pest Management on the Loess Plateau of Ministry of Agriculture, Northwest A&F UniversityYangling, China

**Keywords:** phototaxis, distribution, dopamine, insect behavior, *Acyrthosiphon pisum*

## Abstract

The pea aphid, *Acyrthosiphon pisum* (Harris) (Hemiptera: Aphididae), shows wing polyphenism (winged and wingless morphs) in its life cycle. The winged morph is adapted for dispersal; its two developmental adult stages (for dispersal and reproduction) are based on its breeding periods. The two morphs show different phototactic behavior and the winged can change its preference to light according to the developmental stages. To determine the mechanism and ecological functions of phototaxis for *A. pisum*, we first investigated the phototaxis of the two aphid morphs at different stages and analyzed the phototactic response to lights of different wavelengths; the correlation between alate fecundity and their phototactic behaviors were then studied. Finally, we focused on the possible functions of phototaxis in aphid host location and distribution in combination with gravitaxis behaviors. Negative phototaxis was found for breeding winged adults but all the other stages of both winged and wingless morphs showed positive phototaxis. The reactions of the aphids to different wavelengths were also different. Nymph production in winged adults showed negative correlation to phototaxis. The dopamine pathway was possibly involved in these behavior modifications. We speculated that winged adults can use light for dispersal in the early dispersal stage and for position holding in the breeding stage. Based on our results, we assume that light signals are important for aphid dispersal and distribution, and are also essential for the pea aphids to cope with environmental changes.

## Introduction

The pea aphid *Acyrthosiphon pisum* (Harris), exhibits wing phenotypes at various stages of its life cycle (Braendle et al., 2006). Normally, winged morph is adapted for dispersal and wingless morph for reproduction. The winged and wingless phenotypes in aphids differ in morphology, physiology, and behavior. The winged morph exhibits an elaborate sensory system for flight and host plant location, such as more fully developed compound eyes, ocelli, and longer antennae with more rhinaria as compared with the wingless morph. The wingless morph lacks wings and the wing musculature for dispersal, but it has a faster development time and a larger body size for production than the winged morph (Braendle et al., 2006; van Emden and Harrington, 2007; Brisson, 2010). Density (tactile stimulation) and nutrition (host plant quality) are considered to be the key environmental cues affecting the transformation from winged to wingless morphs. This represents an adaptation of the pea aphid to the environment (Johnson, 1965; Lees, 1967; Sutherland, 1969; Sutherland and Mittler, 1971; Wratten, 1977).

Taxis is the movement of an organism in response to different stimuli including physical, chemical, and biological ones; movements of an organism toward or away from a stimulus, are defined as positive or negative taxis, respectively. Many types of taxis have been identified, such as chemotaxis (by chemicals), electrotaxis (by electric current), gravitaxis (by gravity), hydrotaxis (by moisture), phototaxis (by light), and thermotaxis (by temperature) (Hader, 1987; Mori and Ohshima, 1995; Alon et al., 1999; Pringault and Garcia-Pichel, 2004; Rezai et al., 2010). Phototaxis is a locomotory movement toward or away from a light stimulus for an organism. Many organisms show phototaxis from prokaryotes to eukaryotes including plants and animals (Bulkowski and Meade, 1983; Armitage and Hellingwerf, 2003; Chen et al., 2012). Insects also display such behaviors; for instance, moths, wasps, and whiteflies (Summers, 1997; Castrejon and Rojas, 2010; Chen et al., 2012; Yang et al., 2012). Phototaxis has been widely used in pest control; for instance, in the invention of light traps and yellow card traps, which are based on insect phototaxis to ultraviolet and yellow light (Bowden, 1982). On the other hand, the phototaxis of an organism may change during its life time. It has been recorded that some insect species can change their phototaxis or geotaxis in certain situations, such as developmental stages and starvation (De Ruiter and van der Horn, 1957; Barrett and Chiang, 1967; Ben-Shahar et al., 2003; Gong et al., 2010). Phototactic changes in particular situations may also be occurring in some other animals, such as fish, nudibranch, and stomatopod species (Dingle, 1969; Bulkowski and Meade, 1983; Miller and Hadfield, 1986).

Phototactic behaviors are stimulated by light, and light in different wavelengths may affect phototaxis in insects in different ways. Many insects prefer blue light or ultraviolet, and others can be attracted by green or yellow light. Insects can react to more than one wavelength bands (Coombe, 1981; Yang et al., 2003; Mazza et al., 2010; Yamaguchi et al., 2010). Light with different wavelengths can stimulate visual organs and lead to different reactions in insects (Yokoyama, 2000; Briscoe and Chittka, 2001). Studies show sensors in both ocelli and compound eyes could be functional in phototactic behaviors (Garrey, 1918; Gilbert, 1994; Lazzari et al., 1998). The study of one aphid species *Megoura viciae* labeled some photoperiodic photoreceptors including red-light sensitive photoreceptor-specific proteins (CERN-956) and long-wavelength sensitively photoreceptor-specific proteins (COS-1) in ventral neuropile of protocerebrum and eyes (Gao et al., 1999).

As a complex behavior in insects, phototaxis is controlled by the insect neural system. Previous studies in insects and other animal species showed neurotransmitters (dopamine, serotonin, and so on) played specific roles in phototactic behavior. Dopamine (3, 4-dihydroxyphenethylamine, DA) is an important neurotransmitter and hormone of the catecholamine and phenethylamine families (Joh and Hwang, 1987). Dopamine has been well studied in human and other mammals. Studies reveal that dopamine plays important roles in motor function, reward, learning, aggression, addiction, memory, and some other behaviors in invertebrates, as well as those in vertebrates (Coleman and Neckameyer, 2004; Rauschenbach et al., 2012; Martin and Krantz, 2014). Dopamine is also a key chemical in cuticle maturation (sclerotization and melanization) (Gallot et al., 2010). Researches in *Drosophila* showed that dopamine functioned in phototaxis, while dopamine-deficient flies were also defective in positive phototaxis (Neckameyer et al., 2001; Riemensperger et al., 2011). Serotonin played a key role in phototactic behavior in the honeybee, *Apis mellifera* (Thamm et al., 2010). There is also some evidence in other animals: dopamine could prolong while serotonin could repress positive phototaxis in *Bugula neritina* (Pires and Woollacott, 1997), and serotonin has been reported as negatively modifying phototaxis in a species of crab *Carcinus maenas* (McPhee and Wilkens, 1989).

An understanding of phototaxis would be helpful in studies of aphid dispersal and distribution. Previous studies in many aphid species showed that aphids display phototactic behaviors and normally show a positive preference (Kennedy et al., 1961; Kennedy and Booth, 1963; Hajong and Varman, 2002). During our many years of pea aphid rearing, we found that different aphid morphs did have different phototactic behaviors. The understanding of this changeable phototactic behavior and its underlying mechanism in *A. pisum* reflects the adaptation of *A. pisum* to its ecological conditions.

Gravitaxis response might affect aphid's movement patterns and response for upward climbing on host plants. Only few gravitaxis related studies could be found in *Drosophila melanogaster* (Toma et al., 2002; Armstrong et al., 2006) and there is no gravitaxis receptor researches about aphid to our data. Some studies mentioned aphids exhibited negative gravitaxis (Brunissen et al., 2010; Le Roux et al., 2010) or positive and negative gravitaxis (Pettersson et al., 2007) without further detailed studies. The gravitactic behavior of the pea aphid was then determined.

To determine the underling mechanisms of phototactic behaviors, we first recorded the phototactic response of two morphs of the pea aphid in different nymphal instars to white light. Based on the different phototactic results in winged adults, we analyzed the relationship between phototactic response and fecundity; and then we focused on the phototactic behaviors of selected aphids to different wavelengths of light. We also designed experiments to analyze the possible connections between neurotransmitters (dopamine, octopamine, and serotonin) and aphid phototactic behavior changes. Finally, combining with the results for gravitaxis of the pea aphid, we designed an experiment to study the possible functions of phototaxis in pea aphid host-distribution.

## Materials and methods

### Aphids and plants

A red morph of pea aphid was collected from Lanzhou, Gansu Province, China, and reared on broad bean (*Vicia faba* L., var. “Jinnong”) under a long-day condition (16L: 8D; 20 ± 1°C) for more than 30 generations at the Key Laboratory of Applied Entomology, Northwest A&F University, Yangling, Shaanxi, China. All wingless aphids were reared at a low density (< 30 aphids per 4-week-old plant) for more than three generations before they were used. A high density (30 aphids per 2-weeks old plant seedling) was used to stimulate wing formation. Selected winged aphids were reared at a low density (< 30 aphids per 4-weeks old seedling) before they were used in all subsequent experiments.

### Phototaxis behaviors in different instars of winged and wingless pea aphids

**Figure 1:**
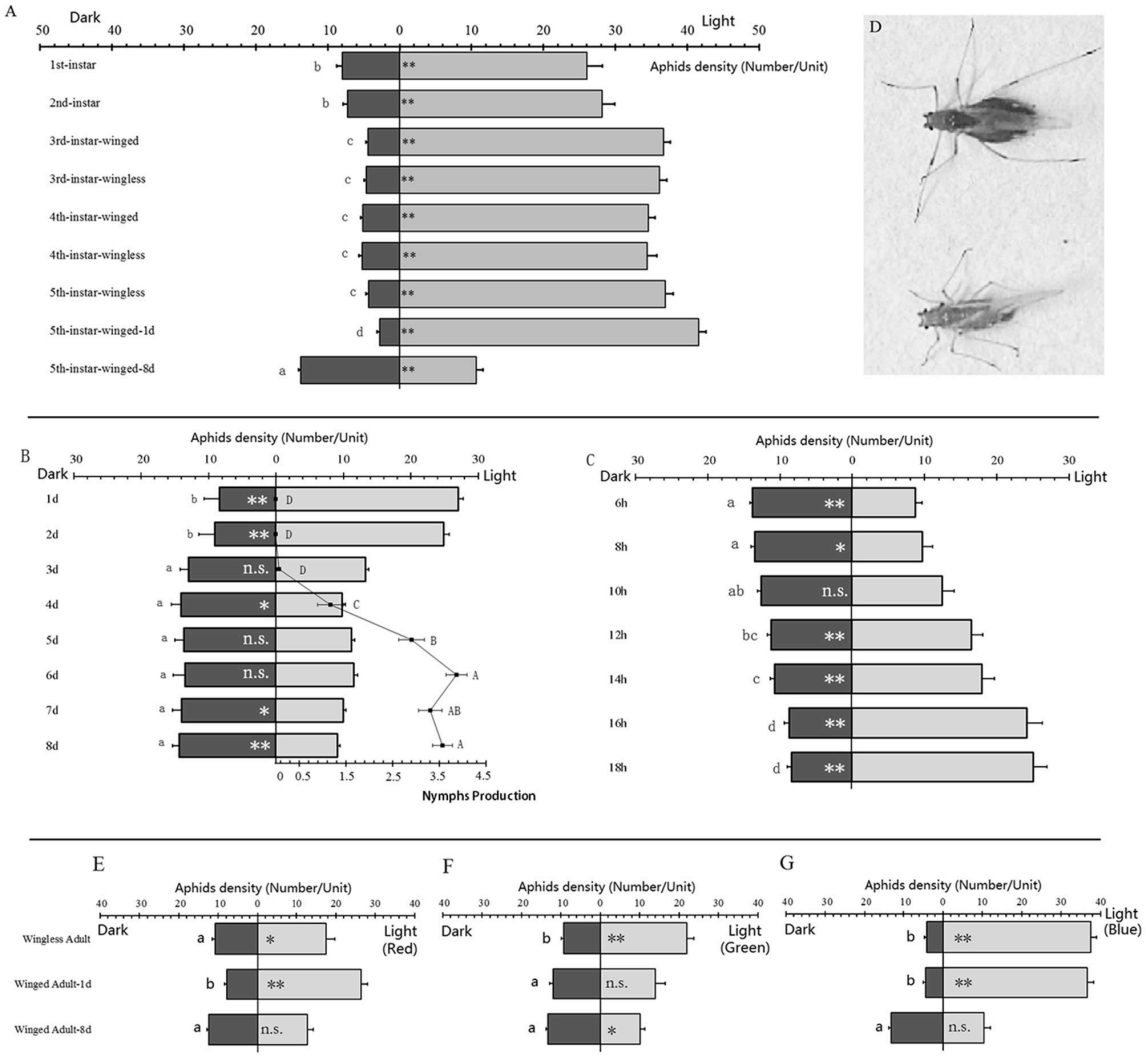
**Phototactic response in different stages and wing forms of *Acyrthosiphon pisum.*** The phototaxis results in different stages of *A. pisum* **(A)**. The fecundity and phototaxis correlations in winged adults during their development **(B)**, and the phototaxis changing in starvation of winged adults in breeding period **(C)**; winged adult in pre-breeding (left) and breeding (right) period **(D)**. Phototaxis in wingless adults 1-day and 8 d winged adults under light with different wavelengths, in red (>600 nm, **E**), green (400–600 nm, **F**) and blue (350–500 nm, **G**). Data were based on aphids' density (number/unit). The light area has 1 unit while dark area has 3 units. * and ** indicate significant different at *P* < 0.05 and *P* < 0.01, respectively (Student's *t*-test). The different letters next to the bars indicate significant differences at *P* < 0.05 (Duncan test).

Phototactic preference of the aphids were determined by comparing the numbers of aphids that moved to a lighted area or remained in a dark area as shown in **Figure 5A**. The arena was made of a transparent plastic petri dish (90 mm in diameter). A lighted area was formed by placing a piece of white filter paper (45 mm in diameter) in the bottom for light reflection; a cold light (KL 1500 LCD, Zeiss, German, set color temperature at 6500 K, 2000 lx) was used as a light source. The lens was focused on the filter paper forming a sharp-edged circle (45 mm in diameter, the same size as the filter paper). The remaining area remained in the dark. The lighted area was one fourth and the dark area was three fourth of the arena. We tested the phototactic preference of wingless nymphs (first and second instars), winged nymphs (third and fourth instars), wingless adults (1-day old), and winged adults (newly emerged and 8-days old, Figure 1D). All aphids were starved for 6 h before they were used. The experiments were undertaken in a dark room at 10:00 a.m., and each treatment lasted for 30 min. During the experiment, 50 starved aphids were placed in each arena, and numbers of aphids moved to the lighted area and those remained in the dark area were counted. Each experiment was repeated 15 times.

### Correlation between nymphs production and phototaxis in the winged adults

Newly emerged winged pea aphid adults were reared at a low density (< 30 individuals per seedling). The nymphs produced were individually counted every day for 8 days. Phototaxis was also determined for 8 days as described above. The correlation of nymphs produced and phototaxis was analyzed for each treatment. Each experiment was repeated 15 times.

### Phototaxis of starved winged adults

Eight-days old winged adults in their reproduction period were starved for 6, 8, 10, 12, 14, 16, or 18 h before they were used in the phototaxis experiments as described above. We selected wingless adults (3 days old), and winged adults (1-day old and 8-days old) in the experiments. Each treatment was repeated 15 times.

### Phototactic behaviors to different light wavelengths

In this experiment, we determined the effects of three light wavelengths on phototaxis of different morphs of the pea aphid. The three wavelengths (red: >600 nm; green: 400–600 nm; blue: 350–500 nm) were obtained by using band filters on the lenses of a cold light source (KL 1500 LCD) (**Figure 5B**). Phototaxis of the aphids was determined as described above. Each treatment was repeated 15 times.

### Transcription in *DDC, TβH,* and *TPH*

Detection of rate-limiting enzyme transcription levels in neurotransmitter production was used for neurotransmitter analysis. Considering the high L-DOPA contents in the host plants *V. faba* (Ingle, 2003; Zhang et al., 2016), we picked *DDC* (DOPA Decarboxylase) downstream for dopamine analysis; *T*β*H* (Tyramine β-Hydroxylase, converted L-tyramine to octopamine) was selected for octopamine and *TPH* (Tryptophan Hydroxylase, and converted L-tryptophan to 5-Hydroxy-L-tryptophan) for serotonin (Figure S2).

**Figure 2:**
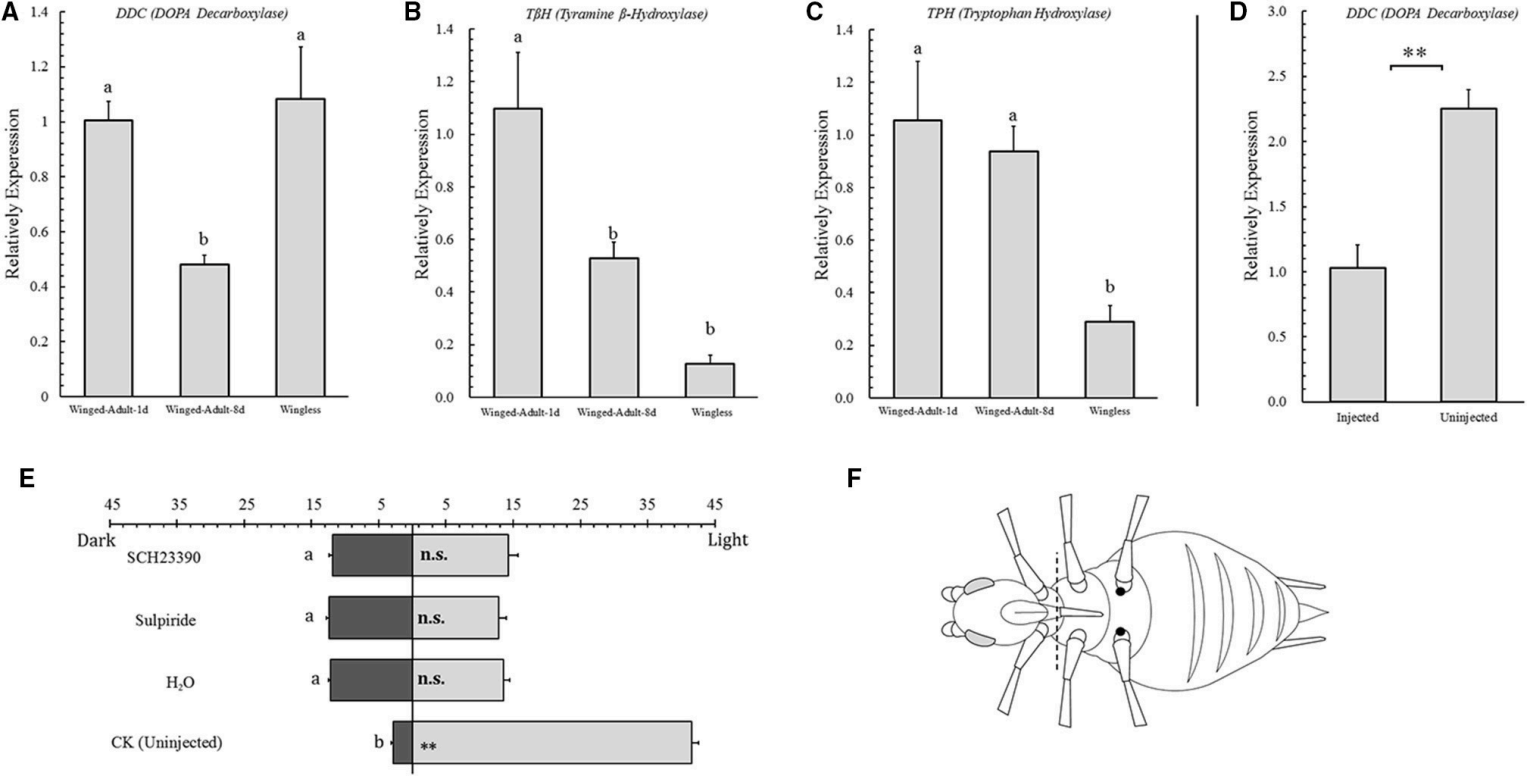
**Relative expression analysis of key enzymes of neurotransmitters in selected *Acyrthosiphon pisum* and phototactic tests after injection experiments in *A. pisum.*** Relative expression levels of *DDC* (DOPA Decarboxylase, **A**), *T*β*H* (Tyramine β-Hydroxylase, **B**) and *TPH* (Tryptophan Hydroxylase, **C**) in three selected *Acyrthosiphon pisum* (1-day and 8-day winged adults and wingless adults) and their phototaxis tests **(E)**; relative expression levels of *DDC* between injected and uninjected *A. pisum* **(D)**. Dissection (dotted line) and injection (dark spots) position are showing in **(F)**. Each value represents the mean ± SEM from independent determinations. ** in both **D** and **E** indicate that the means are significantly different at *P* < 0.01 (Student's *t*-test), and different letters on top of the bars indicate significant differences at *P* < 0.05 (Duncan test).

To analyze expression differences in *DDC, T*β*H,* and *TPH,* wingless and winged adults in the first and eighth day were snap-frozen and dissected between the T1 and T2 segments (Figure 2F). The head and T1 segment (to avoid affecting embryos in the abdomen) were used for transcription tests, and 15 individuals were prepared for one repetition. There experiments were repeated three times. Aphid samples were frozen using liquid nitrogen immediately after collection. RNA was extracted with RNAiso Plus (Takara, Japan), and cDNA was synthesized using a PrimeScript™ RT reagent kit with gDNA Eraser (Takara, Japan). Quantitative real-time PCR (qRT-PCR) was performed with SYBR® Premix Ex Taq™ II (Takara, Japan) in an IQ-5 system (Bio-Rad, Berkeley, California, USA). The primers were designed by Primer-BLAST of NCBI online (http://www.ncbi.nlm.nih.gov/tools/primer-blast/index.cgi?LINK_LOC=BlastHome) (Table S1).

### Dopamine antagonist treatments

To further analyze the dopamine functions in phototactic behavior, dopamine receptor antagonist was injected to modify the dopamine level in the pea aphids. Based on the aphid's locomotion and pretests, 1-day old winged adults were used for the experiments. SCH23390 (R(+)-7-chloro-8-hydroxy-3-methyl-1-phenyl-2, 3, 4, 5-tetrahydro-1H-3-benzazepine hydrochloride, CAS 125941-87-9, sigma, D1 receptor antagonist) (Peczely et al., 2014) and Sulpiride ((−)-5-(aminosulfonyl)-N-[(1-ethyl-2-pyrrodinyl)methyl]-2-methoxy-benzamide, CAS 15676-16-1, sigma, D1 and D2 receptor antagonist) (Hauber and Lutz, 1999) were used in the experiments. A high dose (2.5 mM) of antagonist was used for injection as described by Vergoz et al. (2007).

The methods of injection of dopamine antagonist solution were described by Barron et al. (2007) and Scheiner et al. (2002). A glass needle (P-97 Micropipette Puller, Sutter, CA, USA; a pulling program: Pull = 100, VEL = 100, and Time = 100) attached to the Nanoject II™ Auto-Nanoliter Injector (Drummond Scientific Company, USA) was used in the injection. Dopamine antagonist solution was injected into the thorax (T3 segment) of the aphids, and thoracic injections were made through the fissure at the base of their hind legs (Figure 2F); 200 nl solution was injected per aphid; ddH_2_O in the same dose (200 nl) was used as a control. The aphids were then moved to *V. faba* after treatment. All samples were used for phototaxis tests after 8 h host rearing followed by 6 h starvation. Phototaxis experimental protocol are described above. Injected and control winged aphids (1-day) for *DDC* were used for transcription analysis. The transcription analysis protocol was followed as described above.

### Gravitaxis analysis

#### Bottom release

Fifty aphids were put on the bottom dish of the device and sealed by aluminum foil. The cylinders were divided into five parts by height (A, B, C, D, and E). The experiments lasted for 3 h, and the aluminum foil was then removed, and number of aphids in each position was counted.

#### Top release

Fifty selected aphids were put into the container. The dish with aphids was covered for 1 h; the device was sealed with aluminum foil immediately after aphid release. The experiment lasted for 3 h, and the aluminum foil was then removed, and number of aphids in each position was counted. The gravitaxis of the pea aphids was determined as shown in **Figure 5C**.

### The effect of light on aphid spatial distribution on host plants

This experiment was conducted to determine possible effects of lighting direction on phototactic behavior and spatial distribution of pea aphids on host plants (**Figure 5D**). Four-week-old *V. faba* were used as the host plants. A piece of filter paper made into a cone was used to hold aphids. A cold light source (KL 1500 LCD, Zeiss, German, set color temperature at 6500 K, 8000 lx) was placed on top or bottom of the plant depending on experiment design. A no-light treatment was used as a control. Fifty aphids were used each time. The leaves on the plant were marked from top to bottom as A, B, C, and D (**Figure 5D**), and number of aphids on each leaf was counted 3 h later. The experiment was conducted in a dark room, and each experiment was repeated 10 times.

### Statistical analysis

All experimental data generated were collected and subjected to statistical analysis using Student's *t*-test, and one-way ANOVA; means were separated using Duncan test; Kolmogorov–Smirnov test were used for distribution analysis and Pearson correlation test were used for correlation analysis (SPSS version 22; SPSS Inc., Chicago, IL, USA).

## Results

### Phototactic behavior analysis in different instars of winged and wingless pea aphid

The phototaxis responses in the winged adults showed significant difference. Based on the counts of aphids in the dark and light areas, the aphids were strongly attracted to the light except to the 8-days old winged adults which showed negative phototaxis (*t* = −3.108, *df* = 28, *P* = 0.006; Figure 1A). The 1-day-old winged adult showed the strongest taxis to the light while the first and second instars showed weaker light preference than other positive-phototactic aphids (*F* = 51.682, *df* = 8, 126, *P* < 0.0001, Figure 1A).

### Correlation analysis between nymphs production and phototaxis in winged adult pea aphid

The newly emerged winged adults in the first 2 days after emergence showed significant positive phototaxis (1 d, *t* = −7.502, *df* = 17, *P* < 0.0001; 2 d, *t* = −6.014, *df* = 17, *P* < 0.0001, Figure 1B); and showed slight negative phototaxis in the following 4 days (3 d, *t* = −0.301, *df* = 17, *P* = 0.767; 4 d, *t* = 2.720, *df* = 17, *P* = 0.015, 5 d, *t* = 1.542, *df* = 28, *P* = 0.141; 6 d, *t* = 1.003, *df* = 17, *P* = 0.330, Figure 1B); obvious negative phototaxis were observed at the seventh and eighth days (7 d, *t* = 2.851, *df* = 17, *P* = 0.011; 8 d, *t* = 4.698, *df* = 17, *P* < 0.0001, Figure 1B). There was an increase in nymph production during this process (*F* = 73.579, *df* = 7, 232, *P* < 0.0001, Figure 1B), which was negatively correlated with phototaxis (Pearson *r* = −0.724, *P* = 0.042).

### Changes in phototaxis of winged adults (breeding period) in starvation

Eight-days old winged adults could develop positive phototaxis when staved. This happened after 12 h of starvation (6 h: *t* = 4.698, *df* = 17, *P* < 0.0001; 8 h: *t* = 2.337, *df* = 17, *P* = 0.032: 10 h: *t* = −0.025, *df* = 17, *P* = 0.981: Figure 1C), which could change into significant positive phototaxis (12 h: *t* = −3.171, *df* = 17, *P* = 0.006; 14 h: *t* = −3.924, *df* = 17, *P* = 0.001; 16 h: *t* = −6.747, *df* = 18, *P* < 0.0001; 18 h: *t* = −8.098, *df* = 17, *P* < 0.0001: Figure 1C).

### Phototactic behavior analysis in response to different light wavelengths

The aphids showed different phototactic reactions to different light wavelengths. In the red light treatment (>600 nm), wingless adults showed significant positive phototaxis (*t* = −2.676, *df* = 17, *P* = 0.016), and 1-day old winged adults showed stronger preference for red light (*t* = −9.816, *df* = 17, *P* < 0.0001). Eight-days old winged adults showed no reaction to red light (*t* = −0.253, *df* = 17, *P* = 0.803; Figure 1E). In the green light treatment (400–600 nm), wingless adults also showed significant positive phototaxis (*t* = −6.718, *df* = 17, *P* < 0.0001); 1-day-old winged adults showed no reaction to green light (*t* = −0.695, *df* = 17, *P* = 0.496); while 8-days old winged adults showed significant negative phototaxis (*t* = 2.505, *df* = 17, *P* = 0.023; Figure 1F). In the blue light (350–500 nm) experiment, both wingless adults and 1-day old winged adults showed significant positive phototaxis (wingless adults: *t* = −20.270, *df* = 17, *P* < 0.0001; winged adults: *t* = −18.947, *df* = 17, *P* < 0.0001), and for 8-days old winged adults the difference was not significant (*t* = 1.559, *df* = 17, *P* = 0.137; Figure 1G).

### Dopamine, octopamine, and serotonin analysis

By analyzing rate-limiting enzyme transcription levels of dopamine (*DDC*), we found that 8-day winged adults showed more significant down-regulation than the others (*F* = 7.635, *df* = 2, 6, *P* = 0.022; Figure 2A). In octopamine (*T*β*H*) analysis, wingless adults showed the lowest expression level among all aphid stages and 1-day-old winged adults showed the highest (*F* = 8.385, *df* = 2, 12, *P* = 0.005; Figure 2B). The serotonin analysis (*TPH*) for wingless adults showed more significant down-regulation than for another two aphids (*F* = 8.385, *df* = 2, 9, *P* = 0.010; Figure 2C).

### Dopamine antagonists treatment

After 1-day-old aphid adults were injected, their phototactic behaviors also changed. All injected aphids showed no response to light (SCH23390, *t* = −1.539, *df* = 28, *P* = 0.135; sulpiride, *t* = −0.319, *df* = 28, *P* = 0.752; H_2_O, *t* = −1.296, *df* = 28, *P* = 0.206; Figure 2E).

By analyzing *DDC* transcription level between injected aphids and the control, we found that *DDC* showed significant down-regulation in injected individuals (*t* = −5.334, *df* = 4, *P* < 0.006; Figure 2D).

### Gravitaxis analysis

**Figure 3:**
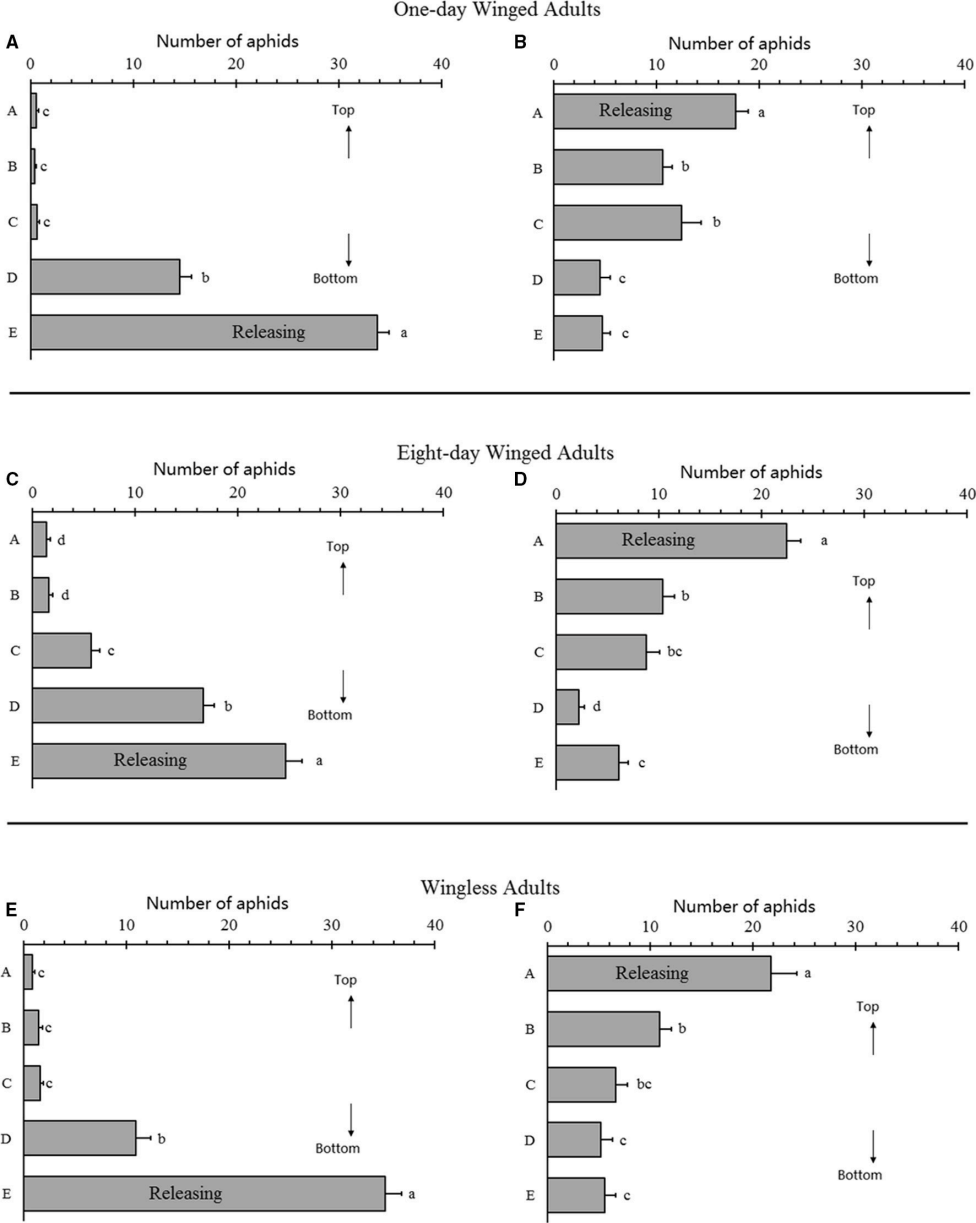
**Gravitaxis analysis of selected *Acyrthosiphon pisum***. One-day winged adults in bottom-releasing **(A)** and top-releasing **(B)**; 8-day winged adults in bottom-releasing **(C)** and top-releasing **(D)**, wingless adults in bottom-releasing **(E)** and top-releasing **(F)**. Different letters next to the bars are significantly different (*P* < 0.05, Duncan test).

Gravitaxis analysis indicated that no obvious gravitaxis in all aphids tested. In the bottom release experiment, all aphids were still at the bottom 3 h after the experiment (1-day-old winged adults, *F* = 357.987, *df* = 4, 70, *P* < 0.0001; 8-days-old winged adults, *F* = 105.846, *df* = 4, 70, *P* < 0.0001; wingless adults, *F* = 213.327, *df* = 4, 70, *P* < 0.0001; Figures 3A,C,E). In the top release experiment, most aphids were still at the top, although some moved downward. One-day-old winged adults showed a wider spread (1-day-old winged adults: *F* = 19.088, *df* = 4, 70, *P* < 0.0001; 8-days-old winged adults: *F* = 46.870, *df* = 4, 70, *P* < 0.0001; wingless adults: *F* = 19.649, *df* = 4, 70, *P* < 0.0001; Figures 3B,D,F), which represented some degree of positive gravitaxis.

### Light attraction analysis in pea aphid host spatial distribution

**Figure 4:**
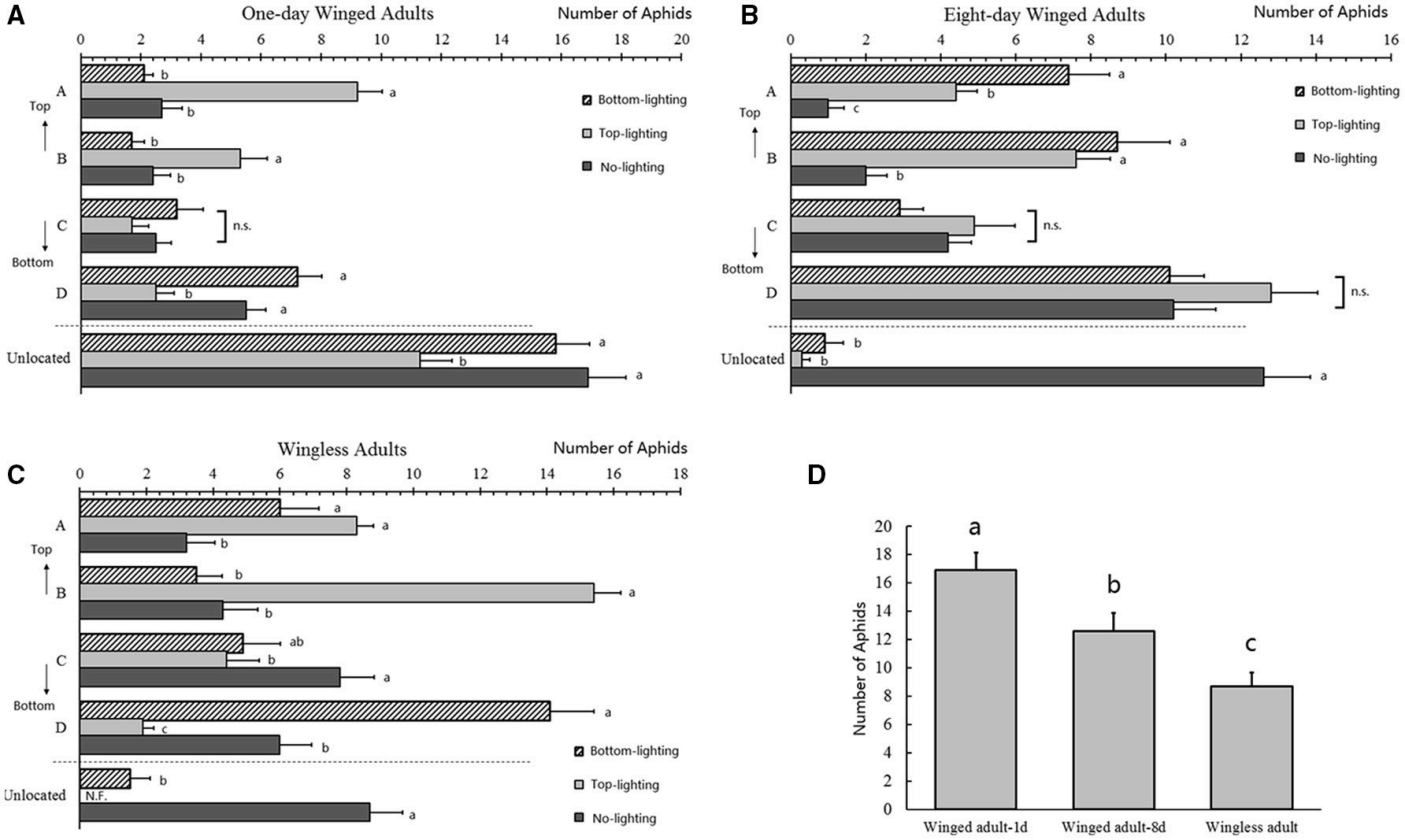
**Distributions of *Acyrthosiphon pisum* under different lights**. Distributions of 1-day old winged adults **(A)**, 8-days old winged adults **(B)**, and wingless adults **(C)** as indicated by the aphids numbers in each part under different lights. Aphid lost in the three selected aphids were shown in **(D)**. Different letters next to the bars are significantly different (*P* < 0.05, Duncan test).

The spatial distribution of 1-day old winged adults was affected by lighting direction, and more aphids aggregated at the lighting source (A: *F* = 8.339, *df* = 2, 27, *P* = 0.001; B: *F* = 57.709, *df* = 2, 27, *P* < 0.0001; C: *F* = 3.065, *df* = 2, 27, *P* = 0.063; D: *F* = 42.685, *df* = 2, 27, *P* < 0.0001). More than one third of the aphids (bottom lighting: 52.67%; top lighting: 37.67%; no lighting: 56.33%) were not on the host after the experiments, and the bottom-lighting attracted more aphids than the top-lighting and no-lighting treatments (*F* = 49.242, *df* = 2, 27, *P* < 0.0001; Figure 4A).

**Figure 5:**
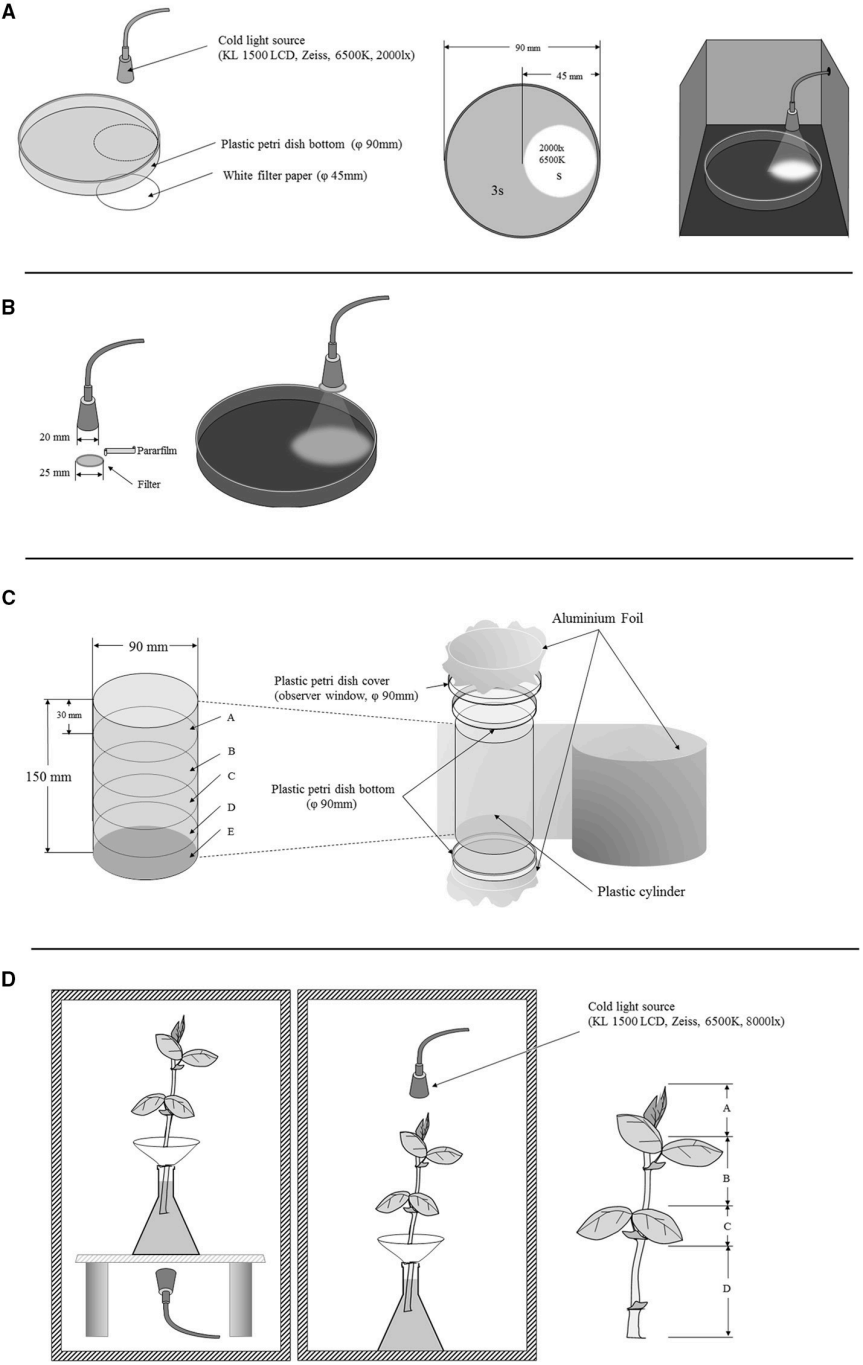
**The set-up of the phototaxis experiment of *Acyrthosiphon pisum*: for phototactic response in different stages and wing forms of *Acyrthosiphon pisum* (A); for phototactic response with different wavelengths (B); for gravitaxis of *A. pisum* (C); and for distributions under different lights (D)**.

For 8-day winged adults, the distribution was also affected by lighting direction (Figure S1A), and only position A (marked on Figure 5D) had a significant increase in aphids numbers in the bottom-lighting treatment (A: *F* = 37.175, *df* = 2, 27, *P* < 0.0001; Figure 4B). No differences were obtained in other positions (B: *F* = 8.157, *df* = 2, 27, *P* = 0.002; C: *F* = 1.287, *df* = 2, 27, *P* = 0.293; D: *F* = 11.532, *df* = 2, 27, *P* < 0.0001; Figure 4B) between the top- and bottom-lighting treatments. However, the aphid distribution was different in the no-light treatment. Less than 3% of aphids were not on plant in the two lighting treatments (bottom lighting: 3%; top lighting: 1%), while 42% failed to find their host without light; the difference was significant (*F* = 6.592, *df* = 2, 27, *P* = 0.005; Figure 4B).

The distribution of wingless adults on hosts was also strongly affected by light (Figures S1A,C). Numbers of aphids were different in positions B and D (marked on Figure 5D); more aphids aggregated at the lighting source (A: *F* = 18.118, *df* = 2, 27, *P* < 0.0001; B: *F* = 12.427, *df* = 2, 27, *P* < 0.0001; C: *F* = 1.586, *df* = 2, 27, *P* = 0.223; D: *F* = 1.917, *df* = 2, 27, *P* = 0.167; Figure 4C). Nearly 29% aphids did not find their host without light, and most or all aphids (bottom lighting: 5%; top lighting: 0%) moved to the plant in the top lighting treatment (*F* = 76.649, *df* = 2, 27, *P* < 0.0001; Figure 4C). Numbers of aphids unable to find their hosts in the no lighting treatments for all treatments. More wingless aphids found their host plants than the winged adults without light (*F* = 12.296, *df* = 2, 27, *P* < 0.0001; Figure 4D).

## Discussion

Our experiments showed that winged *A. pisum* could actually change their phototaxis during their development, and this change can assist aphids in dispersal and host distribution. We found that wingless pea adults showed positive phototaxis while winged adults could change phototaxis from strongly positive to negative depending on their breeding situation. Adults in their reproduction period could regain their phototaxis to positive under starvation. Different morphs showed different reactions to different light wavelengths. We speculated that the dopamine modification pathway was related to phototactic behaviors. These behaviors might assist the aphid to optimize its distribution on host plants

Insects can react to some physical stimuli for environmental adaptation. Phototaxis is one of these behaviors that can be stimulated by light signals (Bowden, 1982; Miller and Hadfield, 1986; Summers, 1997; Briscoe and Chittka, 2001). We found that the pea aphid could also show phototaxis (Figure S1B). The wingless and winged nymphs and wingless adults showed only positive phototaxis (Figure 1A). These results were similar to the studies of Hajong and Varman (2002) on *Sitobion rosaeiformis*. But we also detected that winged adults could change their phototaxis, and these changes were related to their fecundity; the more nymphs they laid the stronger preference for dark they show (Figure 1B). This special behavior appears unique to the winged *A. pisum* adults.

Dopamine and serotonin pathways have been reported in phototaxis modification (McPhee and Wilkens, 1989; Pires and Woollacott, 1997; Neckameyer et al., 2001; Riemensperger et al., 2011). In our investigation, we found that dopamine might be altering phototaxis. Only 8-day-old winged adults showed down-regulation in *DDC,* and they also showed slight negative phototaxis. Expression levels of *DDC* in 1-day-old winged adults and wingless adults were relatively higher than 8-day-old winged adults, and both of them represented positive phototaxis (Figures 1A, 2A). It exhibited a connection between *DDC* expression and phototaxis. This provided similar results to those in *D. melanogaster* and *B. neritina* which high dopamine can increase positive phototaxis (Pires and Woollacott, 1997; Neckameyer et al., 2001; Riemensperger et al., 2011). We assumed that dopamine pathway would function similarly in *A. pisum* as well, and the dopamine pathway downstream (dopamine receptors and dopamine re-uptaking) also need to be studied. In the meantime, we assumed that the octopamine (based on *T*β*H* expression) might represent the locomotion abilities in the pea aphids (dispersal winged adult > breeding winged adult > wingless adult), which dispersal winged morphs showed higher regulation than the other two morphs we selected. Serotonin (based on *TPH* expression) exhibited differences between the wingless aphid and the two winged forms (Figures 2B,C), and the mechanism behind needs to be investigated. Previous studies reported that serotonin could also modify phototaxis (McPhee and Wilkens, 1989; Thamm et al., 2010), but in our experiments we observed no connection between serotonin and phototaxis in the pea aphids (Figures 1A, 2C).

We attempted to reveal the dopamine receptors functions in phototaxis by using dopamine antagonist. The treatment was relatively unsuccessful and no differences were observed in our experiment. But to our surprise, all treated aphids showed a weak response to light. We have noticed that injection (physical injury) could affect dopamine production (*DDC* expression). It could lead to declining dopamine levels in all injected aphids (aphids feed on the fluid in plant phloem. Due to the difficulty to feed the aphids dopamine antagonists, our results showed that artificial diet (poor nutrition) could also affect dopamine pathway) (Figure 2D). Both of our *DDC* expression detection of injected and control aphids showed down-regulation, which means all the treated aphids had a decline in dopamine level. This result also supported our assumption regarding the function of dopamine in aphid phototaxis, and the weak negative phototaxis that observed might be caused by dopamine biosynthesis declining. But the multiple functions of dopamine revealed that phototactic behavior is not likely to be simple, and we still need a better way to obtain precise results.

Considering that the winged adult was the only morph that responded to population dispersal and its strong response to light, the changes in phototactic behaviors would affect their movement patterns in winged *A. pisum*. It is reasonable that the negative phototactic behavior of breeding winged aphids has a special ecological function that related to light signal, and aphids' movement patterns (host distribution) in host was considered as a breakthrough. The results in the spatial distribution of the aphids on plants indicated that positive phototaxis of the wingless aphids could reach plant top. If the light source changed downward, their distribution could be affected. Because the direction of sunlight is normally from above, we assumed that sunlight could be a beacon for aphids to identify where the host top is located and to move toward it. For winged aphids, we believe that the multiple functions of phototaxis could assist other behaviors. Combing with our studies, we hypothesized that in the early stage of winged adults (newly emerged, pre-breeding), strong positive phototaxis could help the aphids reach the top of a plant and fly away. After they found a host and started to develop into breeding winged morphs, positive phototaxis would be unwise, and the aphids need to find a way to keep themselves sedentary. Therefore, based on our results, we assumed that an altering phototaxis could help in solving this problem. On the other hand, if the host was not familiar to the aphids in wild condition, negative phototaxis could help them to walk downward and get away from the immature part (buds and new leaves), which probably have a stronger resistance to aphids or being predated by nature enemies. Starvation normally represents a lack of nutrition, and long duration of starvation could reverse phototaxis, which may lead aphids back to the top position in order to fly away in our opinion. Changeable phototaxis is common in other animals, representing a flexible adaptation to the environment (De Ruiter and van der Horn, 1957; Barrett and Chiang, 1967; Dingle, 1969; Bulkowski and Meade, 1983; Miller and Hadfield, 1986; Ben-Shahar et al., 2003; Gong et al., 2010). Our researches revealed that *A. pisum* had a similar physiological character. The hypothesis of its ecological functions need more evidences.

Previous studies by Hajong and Varman (2002) in *S. rosaeiformis* also indicated the involvement of phototaxis in aphid distribution, but this aphid species could reach the host bud without light. We found surprisingly that our tested *A. pisum* aphids displayed a strong decline in host finding abilities without lighting, and compared with the two winged individuals, wingless aphids showed better host finding ability (Figure 4).

All aphid in the experiment slightly displayed positive gravitaxis (Figure 3). These results showed the importance of light (or vision) in host location for pea aphid. It has been discussed in earlier studies, vision of the insects may be even more important than olfaction in host finding (Reeves et al., 2009; Reeves, 2011; Machial et al., 2012), and our work also supports this finding.

In conclusion, different pea aphid morphs could display different phototaxis to light stimuli, and light in different wavelength bands could also give different stimuli to aphids. We noticed that light in short wavelengths produced strong positive or negative effects on aphids, and light in middle or long wavelength bands had weaker or no effects on them. The tested aphid morphs showed different reactions to specific light wavelengths. Based on previous studies of aphid's vision organs, different eye constructions (compound eyes, and ocelli missing) might be responsible for the differences in reaction to light (Braendle et al., 2006; Brisson, 2010). Dopamine, a neurotransmitter, might play a certain rule between light signal detection and behavioral response in pea aphid.

**Figure 6:**
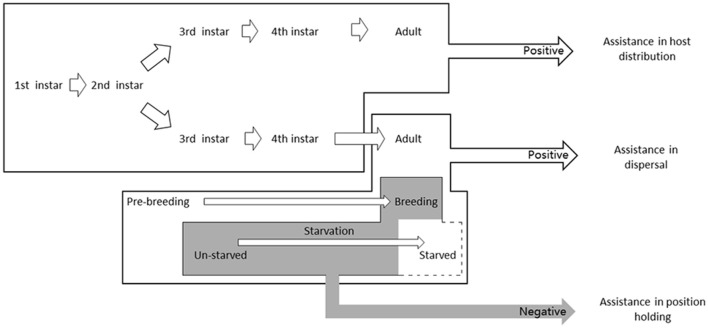
**The hypothesis of phototaxis functions in different *Acyrthosiphon pisum* morphs**. Winged aphids can change their phototactic responses, which help them to disperse during their pre-breeding period and to prevent them to fly away from the host plants during their reproduction period; for the wingless morphs, their positive phototaxis could assist them in locating best parts of host plants.

Winged pea aphids can changes their phototactic responses, which help them to disperse during their pre-breeding period and to prevent them to fly away from the host plants during the period of their reproduction. For the wingless morphs, their positive phototaxis could assist them in locating hosts (Figure 6). We found that the aphids were observed almost unable to locate their host without light so that light signals are extremely important for aphid dispersal and distribution, representing a significant adaptation to ecological environments.

## Author contributions

YZ and TL designed the research; YZ and XW performed research; XJ and HT provided assistance; YZ, XW, and HT analyzed data; YZ and XW wrote the manuscript; XJ, HT, and TL edited the manuscript; and XW and YZ revised the manuscript.

### Conflict of interest statement

The authors declare that the research was conducted in the absence of any commercial or financial relationships that could be construed as a potential conflict of interest.
