# Supplementary material for: Winged Pea Aphids Can Modify Phototaxis in Different Development Stages to Assist Their Host Distribution
Source: Front Physiol. 2016 Aug 2;7:307. doi: 10.3389/fphys.2016.00307 (PMC4969297; doi:10.3389/fphys.2016.00307)

Pea aphid alate can modify phototaxis in different development stages to assist their host distribution

Yi Zhang, Xing-Xing Wang, Xiang-Feng Jing, Hong-Gang Tian, Tong-Xian Liu

Table. S1. Primers used for quantitative PCR

| Genes | ACYPI Identifier | Forward primer | Reverse primer |
| --- | --- | --- | --- |
| *DDC* | ACYPI009626 | CTGACATACTGAGCGATTCA | TCTGGCAAGCCTATCATTTT |
| *TβH* | ACYPI007242 | TCATTACAGCACGCATTTTC | GCTGAATCCTCCAAGTGTAA |
| *TPH* | ACYPI000175 | GTTACCGAGAGGACAACATT | TCATATACTGCGTGCAATGA |

Fig. S1. Winged (1d) and wingless adult *Acyrthosiphon pisum* distribution in *Vicia faba* with top-lighting (A); the reaction of 1d winged adults, 8 days winged adults and wingless adults to light (B); the device for distribution experiments and wingless adults distribution in bottom lighting treatment (C).


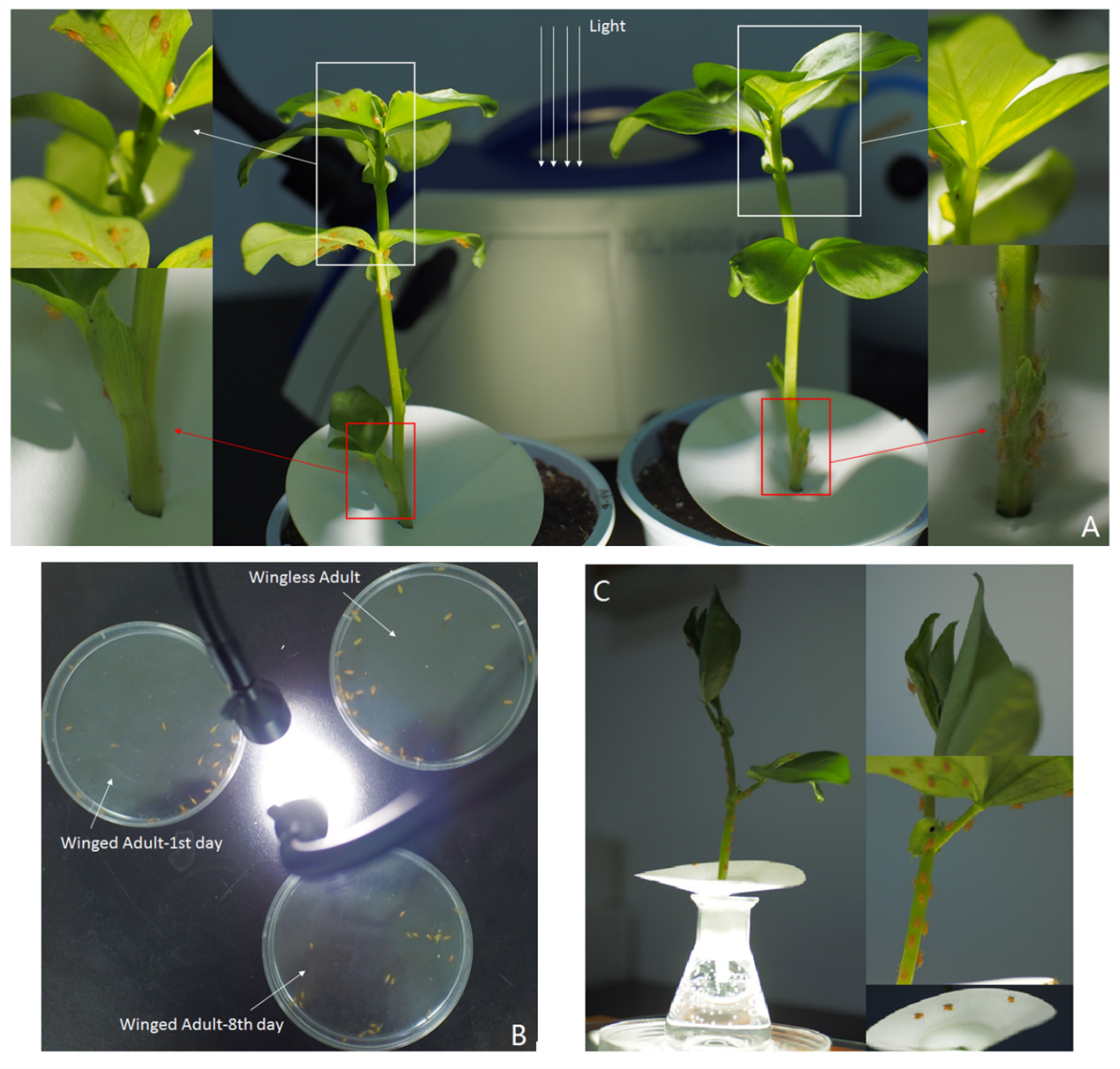


Fig. S2. Biosynthetic pathways and key enzymes of dopamine (A), octopamine (B) and serotonin (C). Selected enzymes are marked in red. Considering the high L-DOPA contents in host plants *V. faba* (Ingle 2003), we picked DDC (DOPA Decarboxylase) downstream for dopamine analysis instead of TH (Tyrosine Hydroxylase, key enzyme of dopamine synthesis).


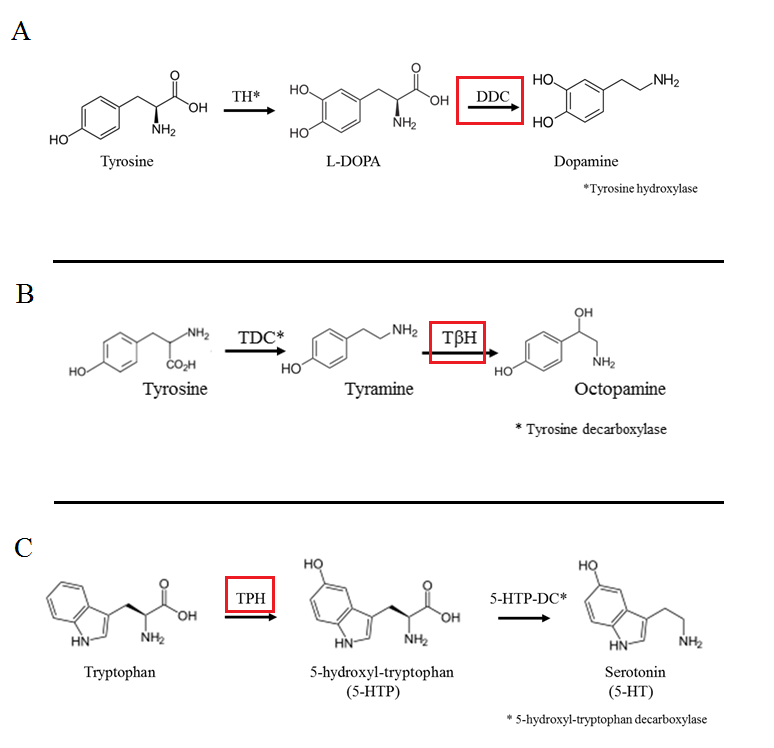

Supplement: Supplementary file 1 [file DataSheet1.DOCX]
